# Supplementary material for: Starting to have sexual intercourse is associated with increases in cervicovaginal immune mediators in young women: a prospective study and meta-analysis
Source: eLife. 2022 Oct 25;11:e78565. doi: 10.7554/eLife.78565 (PMC9596159; doi:10.7554/eLife.78565)
Supplement: Supplementary file 2. — The search string used to query PubMed to identify potentially relevant articles for the systematic review. [file elife-78565-supp2.docx]

## Supplemental File 2 – Systematic review search terms

(("Immunoproteins"[Mesh] OR "Cytokines"[Mesh] OR "Antimicrobial Cationic Peptides"[Mesh] OR "Immunoassay"[Mesh] OR immunoassay*[tiab] OR cytokine*[tiab] OR interleukin*[tiab] OR immunoprotein*[tiab] OR “immune mediator”[tiab] OR “immune mediators”[tiab] OR “immune biomarker”[tiab] OR “immune biomarkers”[tiab] OR “immune modulator”[tiab] OR “immune modulators”[tiab] OR “immune determinants”[tiab] OR “immune environment”[tiab] OR “immune microenvironment”[tiab] OR complement[tiab] OR immunoglobulin*[tiab] OR antibod*[tiab] OR chemokine* OR interferon* OR lymphokine* OR monokine* OR “tumor necrosis factor” OR “tumor necrosis factors” OR “transforming growth factor” OR “transforming growth factors” OR “antimicrobial peptides” OR “antimicrobial peptide” OR “antimicrobial polypeptide” OR “antimicrobial polypeptides” OR defensin OR defensins)

AND

("Vagina"[Mesh] OR "Cervix Uteri"[Mesh] OR vagina*[tiab] OR cervicovaginal[tiab] OR “cervico vaginal”[tiab] OR cervix[tiab] OR cervical[tiab] OR endocervi*[tiab] OR ectocervi*[tiab] OR softcup[tiab] OR “weck cel”)

AND

(“sexually inactive”[tiab] OR “sexual debut*”[tiab] OR “first sex*”[tiab] OR “first intercourse”[tiab] OR “initiating sex*”[tiab] OR “sexual initiation”[tiab] OR “initiate sex*”[tiab] OR virgin[tiab] OR virgins[tiab] OR virginal[tiab] OR “sexually inexperienced”[tiab] OR “sexually abstinent”[tiab] OR “became sexually active”[tiab] OR “never sexually active”[tiab] OR “no history of sexual intercourse”[tiab] OR “never having had sex”[tiab]))

NOT

(“animals”[mh] NOT “humans”[mh])
