# Supplementary material for: Starting to have sexual intercourse is associated with increases in cervicovaginal immune mediators in young women: a prospective study and meta-analysis
Source: eLife. 2022 Oct 25;11:e78565. doi: 10.7554/eLife.78565 (PMC9596159; doi:10.7554/eLife.78565)
Supplement: Supplementary file 3. — The scale used to assess the risk of bias in studies included in the meta-analysis. This scale was custom modified from the Newcastle-Ottawa Quality Assessment scale. [file elife-78565-supp3.docx]

Supplemental File 3 – Risk of bias assessment scale

**MODIFIED** **NEWCASTLE - OTTAWA QUALITY ASSESSMENT SCALE**

**Risk of bias assessment of pre/post-first sexual intercourse studies**

Note: A study can be awarded a maximum of one star for each numbered item within the Selection and Outcome categories. A maximum of two stars can be given for Comparability

**Selection**

1) Representativeness of the pre-first sex cohort

a) truly representative of the average AGYW in the community **🟑**

b) somewhat representative of the average AGYW in the community **🟑**

c) selected group of users eg nurses, volunteers

d) no description of the derivation of the pre-first sex cohort

2) Selection of the post-first sex cohort

a) drawn from the same community as the exposed cohort **🟑**

b) drawn from a different source

c) no description of the derivation of the post-first sex cohort

3) Ascertainment of pre/post-first sex status

a) self report plus markers of sexual exposure (PSA, y-chromosome, pregnancy, STI, or similar) **🟑**

b) self report alone

c) no description

**Comparability**

1) Comparability of cohorts on the basis of the design or analysis

a) study controls for bacterial vaginosis **🟑**

b) study controls for age **🟑**

c) study controls for any additional relevant factor **🟑**

**Outcome**

1) Method of measurement of immune mediator concentrations

a) same method used for both cohorts **🟑**

b) different methods used for each cohort

c) no description

2) Outcome measurements are available for all participants

a) yes **🟑**

b) no but exclusions are unlikely to introduce bias **🟑**

c) no and exclusions may introduce bias

d) no description
