# Supplementary material for: Starting to have sexual intercourse is associated with increases in cervicovaginal immune mediators in young women: a prospective study and meta-analysis
Source: eLife. 2022 Oct 25;11:e78565. doi: 10.7554/eLife.78565 (PMC9596159; doi:10.7554/eLife.78565)
Supplement: Supplementary file 4. — Raw concentration data plots and forest plots for every immune mediator included in the meta-analysis section. The concentration plots show the measured immune mediator concentration for every sample in every study on a log10 scale, with samples from the same individual connected by lines. The forest plots show the effect size and weight in each study as well as the overall meta-estimate. [file elife-78565-supp4.pdf]

Supplemental File 4: Meta-analyses for each immune factor

Log2-fold difference between pre and post-first sex

This section shows the meta-analyses for each immune factor where the concentrations of at least half of the samples fell above the limit of detection. A different immune factor is shown on each page.

Each row represents a different study, with the squares indicating the mean and the lines indicating the 95% confidence intervals. Positive numbers indicate higher concentrations post-first sex, while negative numbers indicate higher pre-first sex. The size of the squares is proportional to how heavily the study is weighted in the meta-analysis.

The center of the diamond and the vertical dotted line indicates the meta-effect as determined by the random effects model. The width of the diamond indicates the 95% confidence interval of the meta-effect.

TE, treatment effect (log2-pg/mL of the post-first sex minus log2-pg/mL of the pre-first sex samples); seTE, standard error of the treatment effect; 95%-CI, 95% confidence interval around the treatment effect; Weight, the percentage of the meta-estimate contributed by each study.

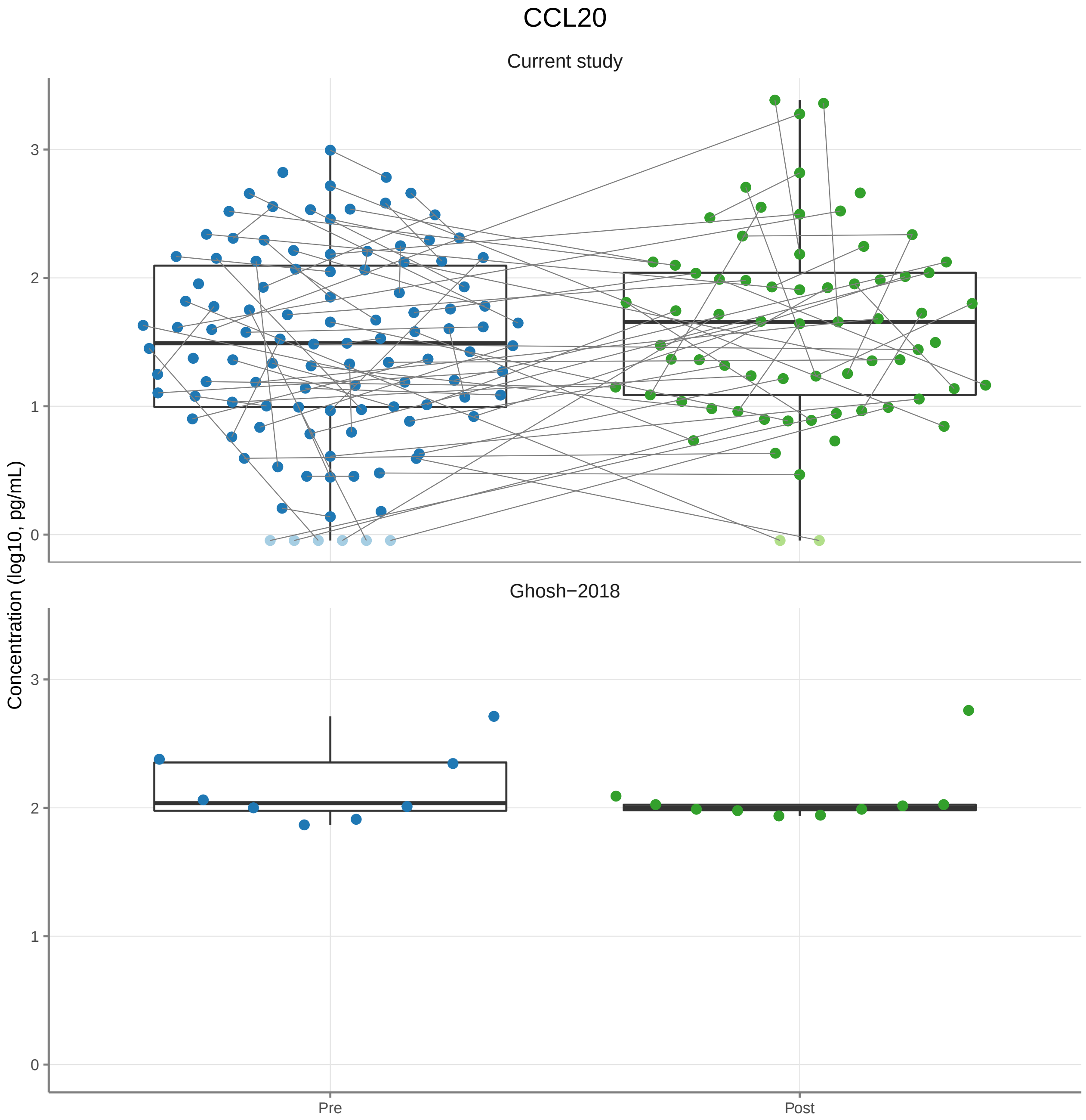

CCL20
Current study
Ghosh−2018
Concentration (log10, pg/mL)
0
1
2
3
Pre
Post

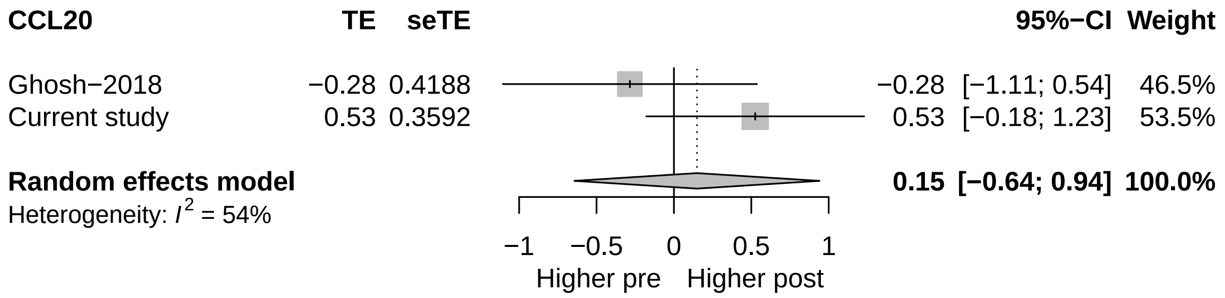
CCL20
TE
seTE
95%−CI
Weight
Ghosh−2018
−0.28
0.4188
−0.28
[−1.11; 0.54]
46.5%
Current study
0.53
0.3592
0.53
[−0.18; 1.23]
53.5%
Random effects model
0.15
[−0.64; 0.94]
100.0%
Heterogeneity: I2 = 54%
−1
−0.5
0
0.5
1
Higher pre
Higher post

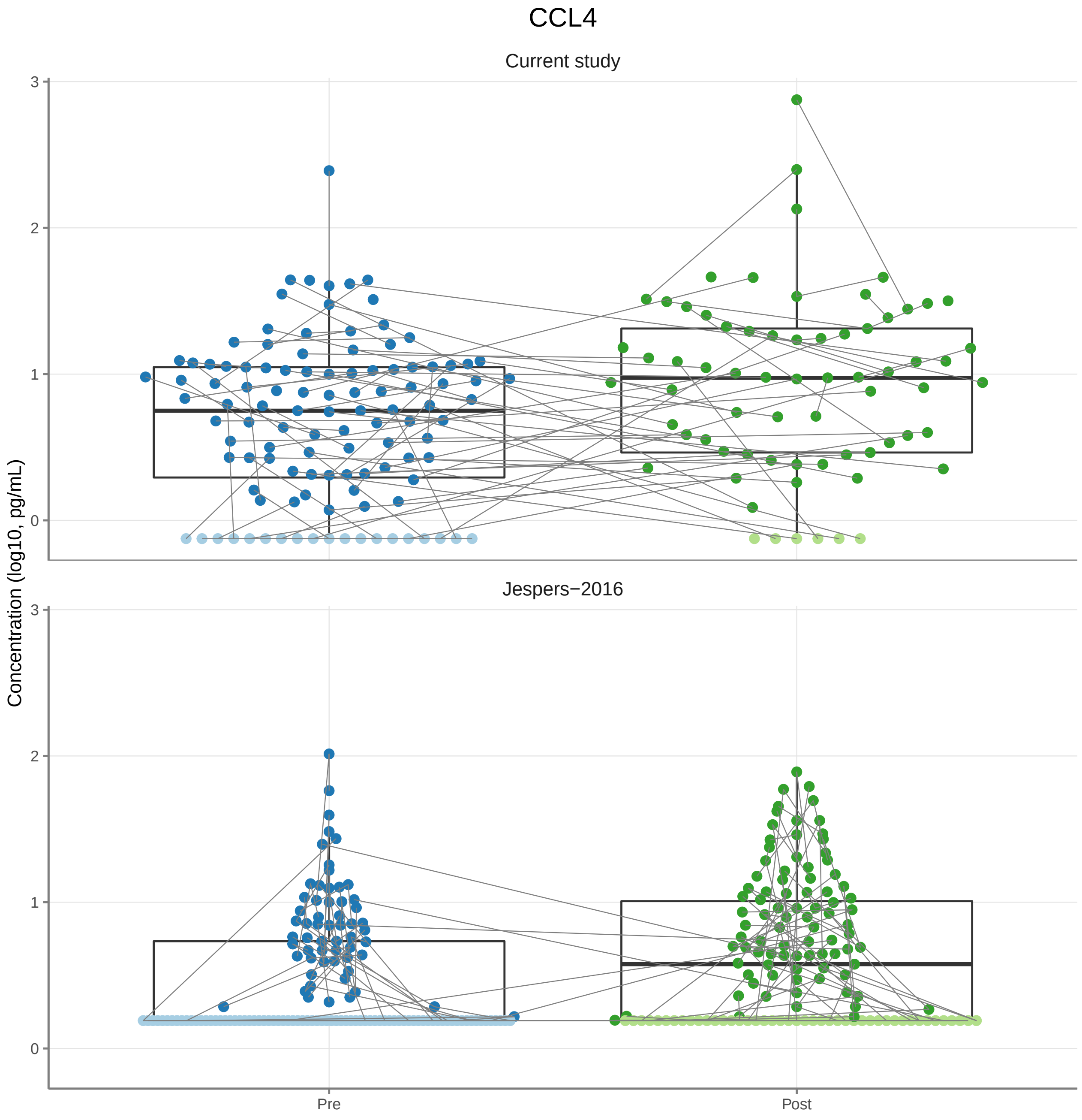
CCL4
Current study
Jespers−2016
Concentration (log10, pg/mL)
3
2
1
0
Pre
Post

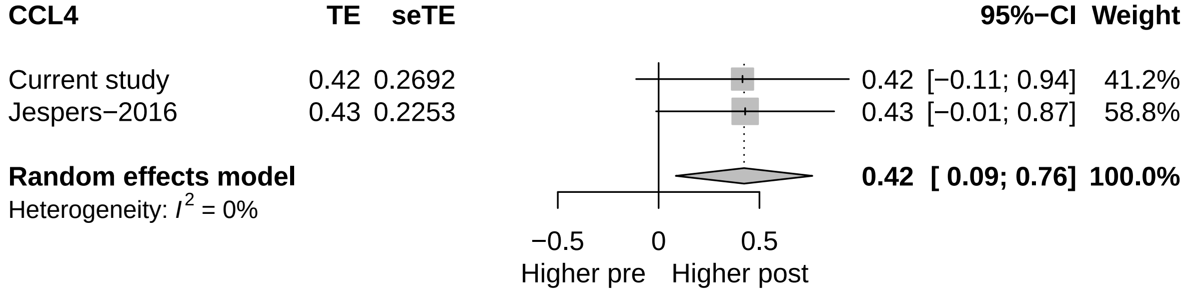
CCL4
TE
seTE
95%−CI
Weight
Current study
0.42
0.2692
0.42 [−0.11; 0.94]
41.2%
Jespers−2016
0.43
0.2253
0.43 [−0.01; 0.87]
58.8%
Random effects model
0.42 [0.09; 0.76]
100.0%
Heterogeneity: I2 = 0%
−0.5
0
0.5
Higher pre
Higher post

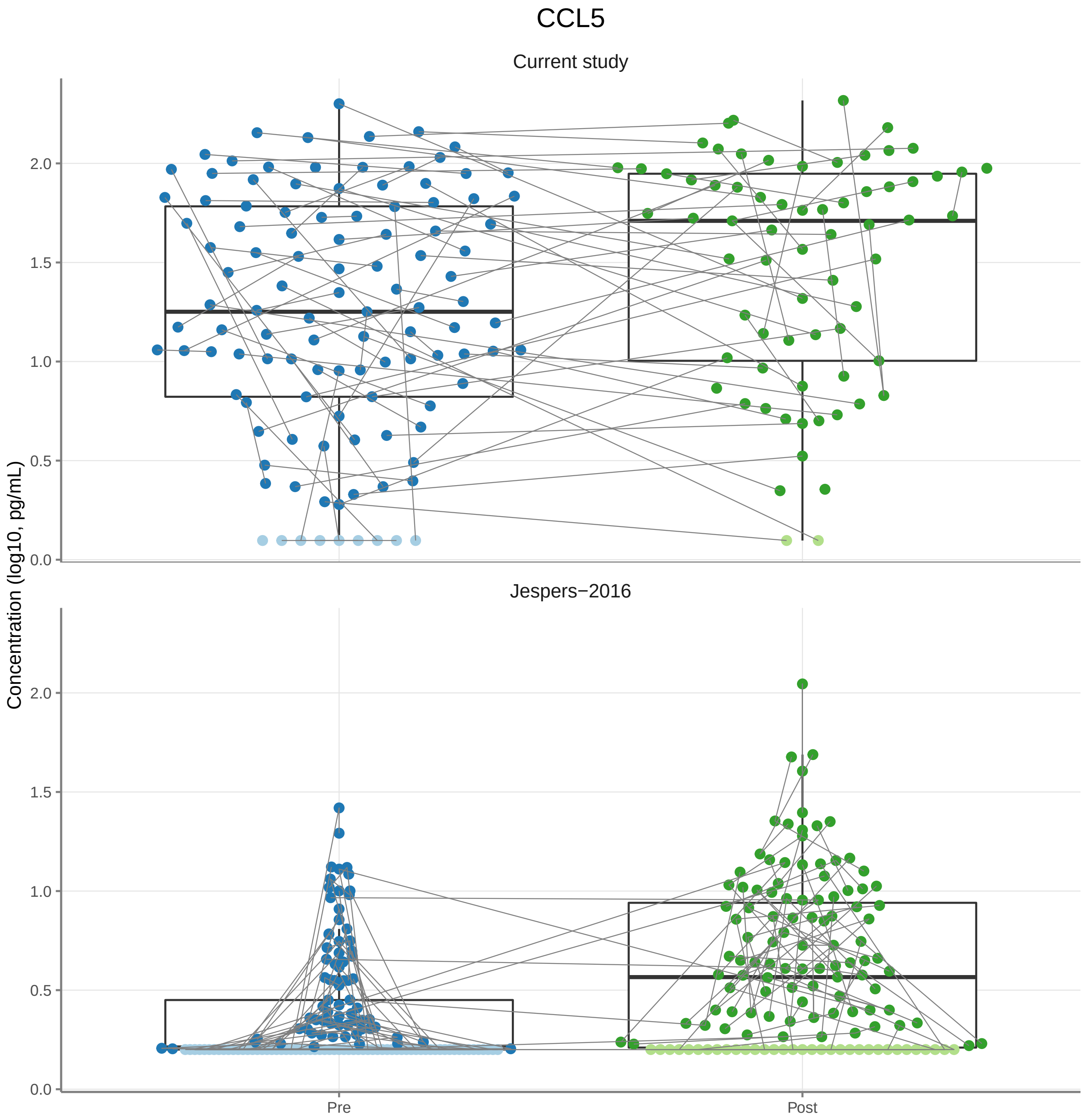

CCL5
Current study
Jespers−2016
Concentration (log10, pg/mL)
2.0
1.5
1.0
0.5
0.0
Pre
Post

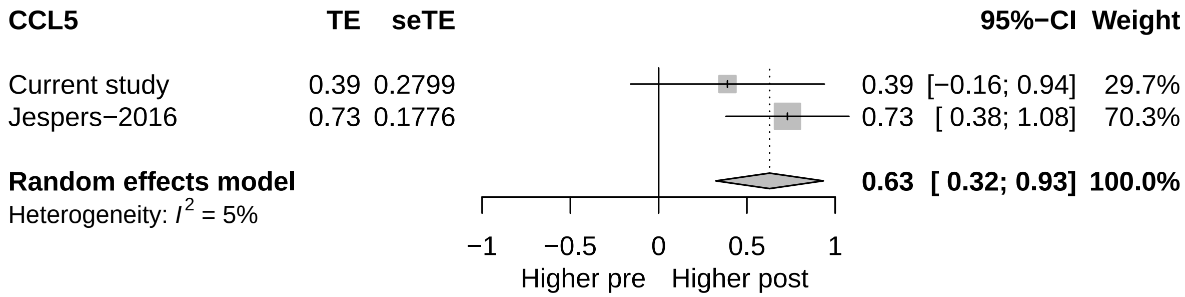
CCL5
TE
seTE
95%−CI
Weight
Current study
0.39
0.2799
0.39 [−0.16; 0.94]
29.7%
Jespers−2016
0.73
0.1776
0.73 [0.38; 1.08]
70.3%
Random effects model
0.63 [0.32; 0.93]
100.0%
Heterogeneity: I2 = 5%
−1
−0.5
0
0.5
1
Higher pre
Higher post

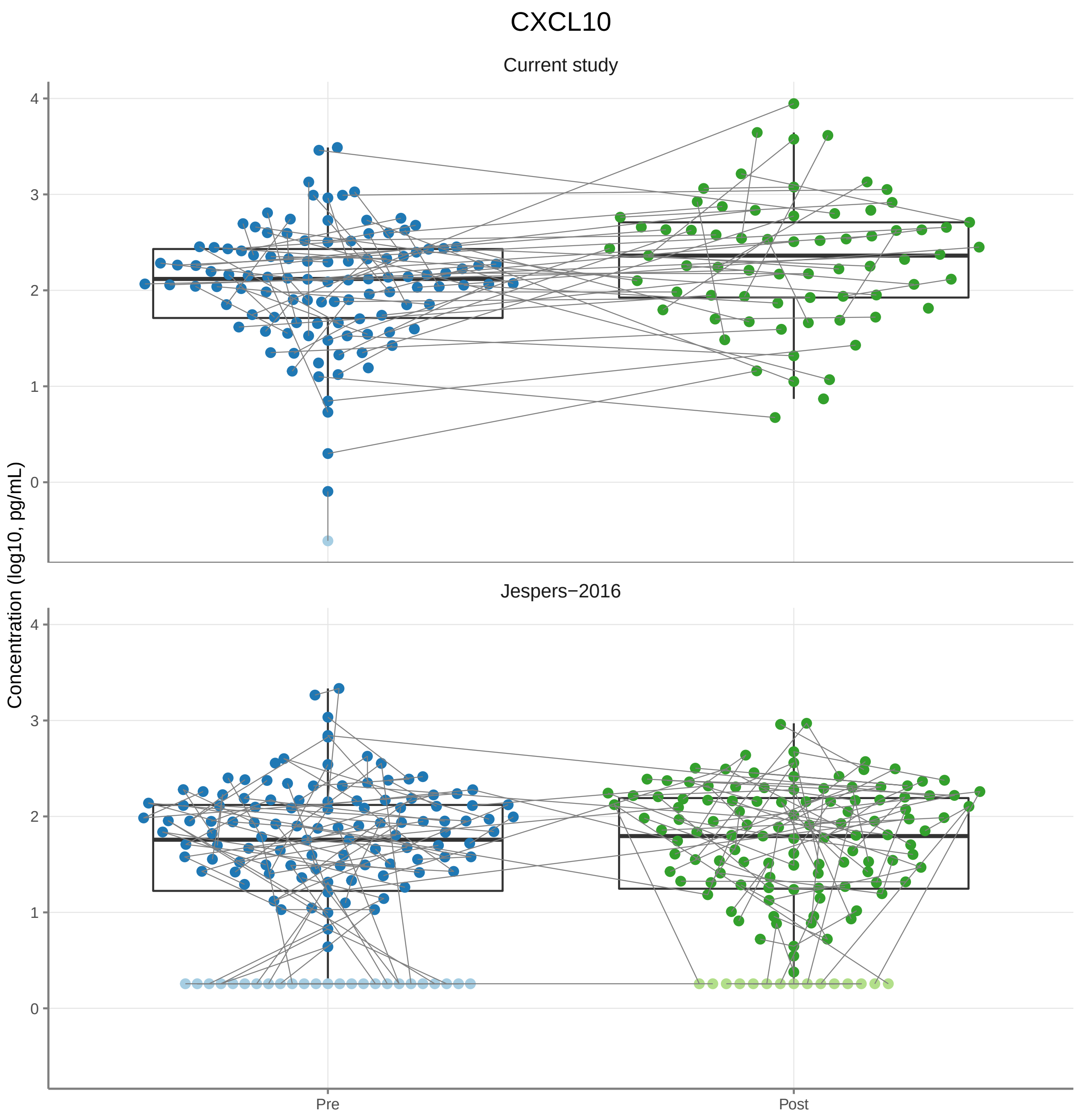
CXCL10
Current study
Jespers−2016
Concentration (log10, pg/mL)
4
3
2
1
0
Pre
Post

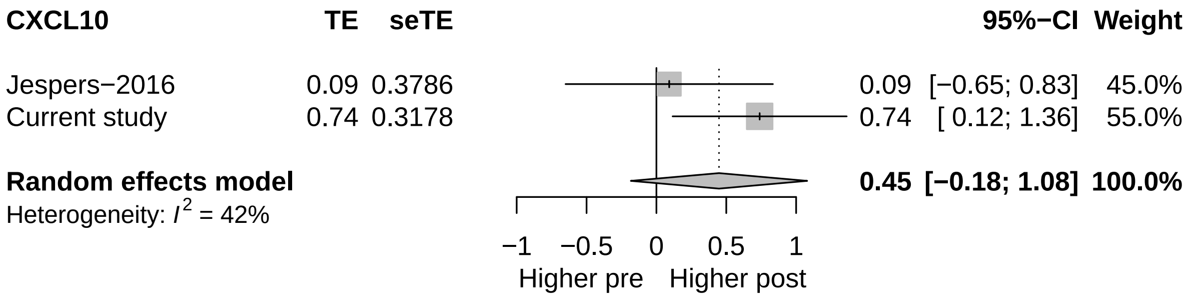
CXCL10
TE
seTE
95%−CI
Weight
Jespers−2016
0.09
0.3786
0.09
[−0.65; 0.83]
45.0%
Current study
0.74
0.3178
0.74
[0.12; 1.36]
55.0%
Random effects model
0.45
[−0.18; 1.08]
100.0%
Heterogeneity: I2 = 42%
−1
−0.5
0
0.5
1
Higher pre
Higher post

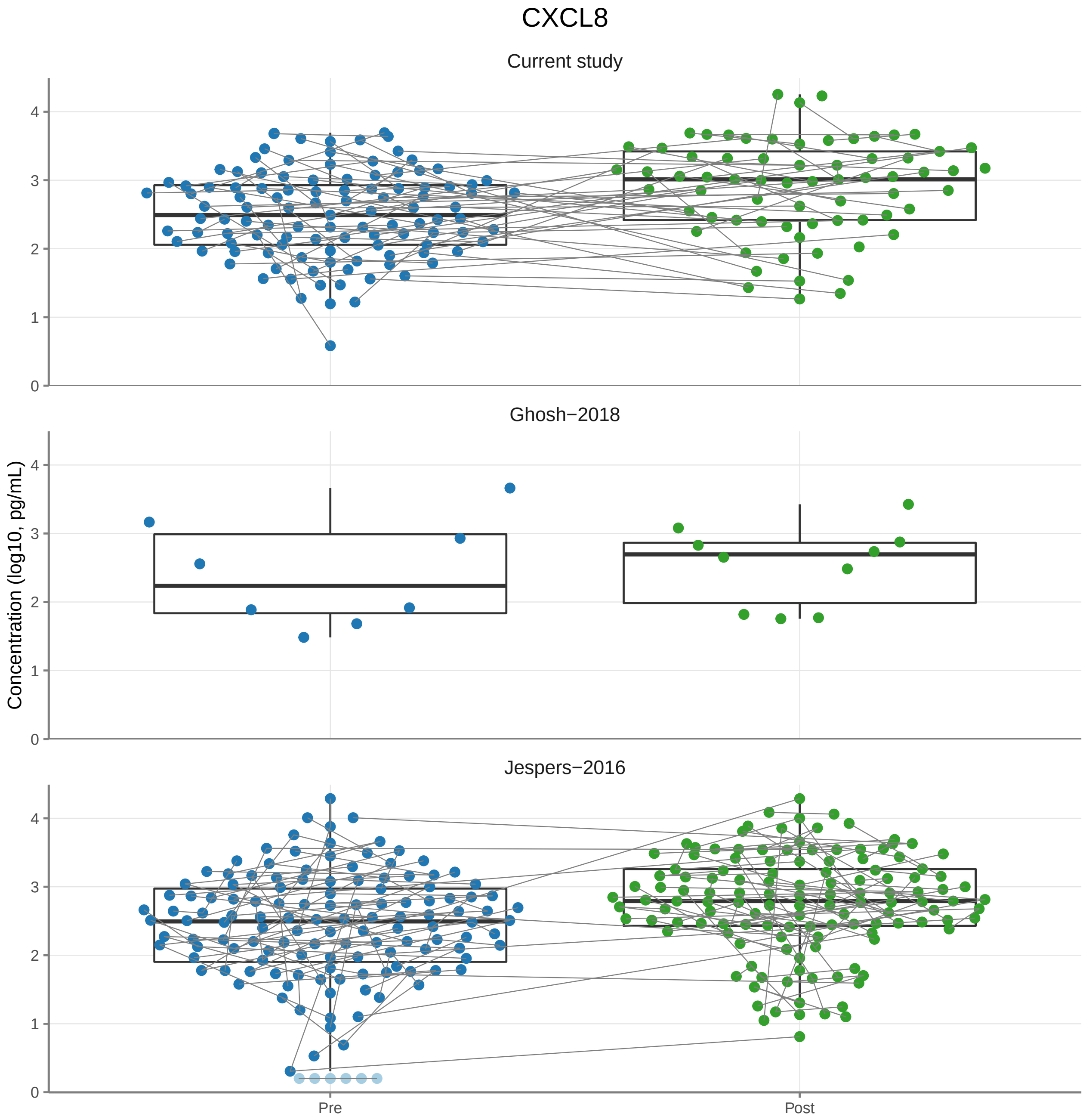

CXCL8
Current study
Ghosh−2018
Jespers−2016
Concentration (log10, pg/mL)
0
1
2
3
4
Pre
Post

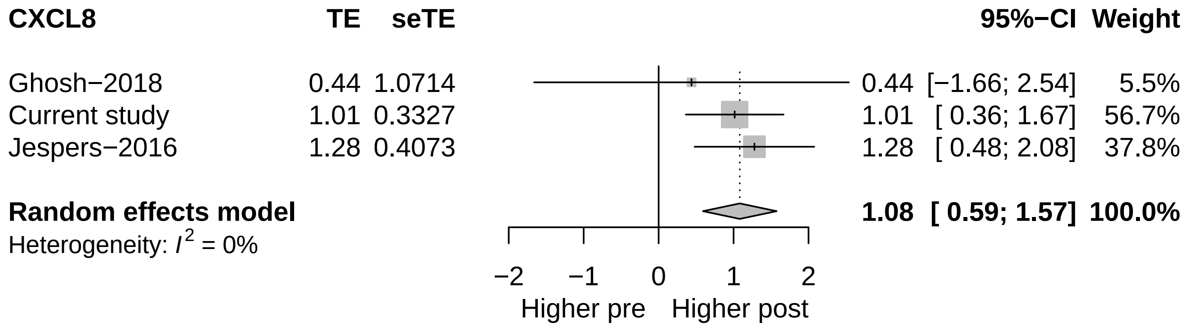
CXCL8
TE
seTE
95%−CI
Weight
Ghosh−2018 0.44 1.0714 0.44 [−1.66; 2.54] 5.5%
Current study 1.01 0.3327 1.01 [0.36; 1.67] 56.7%
Jespers−2016 1.28 0.4073 1.28 [0.48; 2.08] 37.8%
Random effects model 1.08 [0.59; 1.57] 100.0%
Heterogeneity: I2 = 0%
−2
−1
0
1
2
Higher pre
Higher post

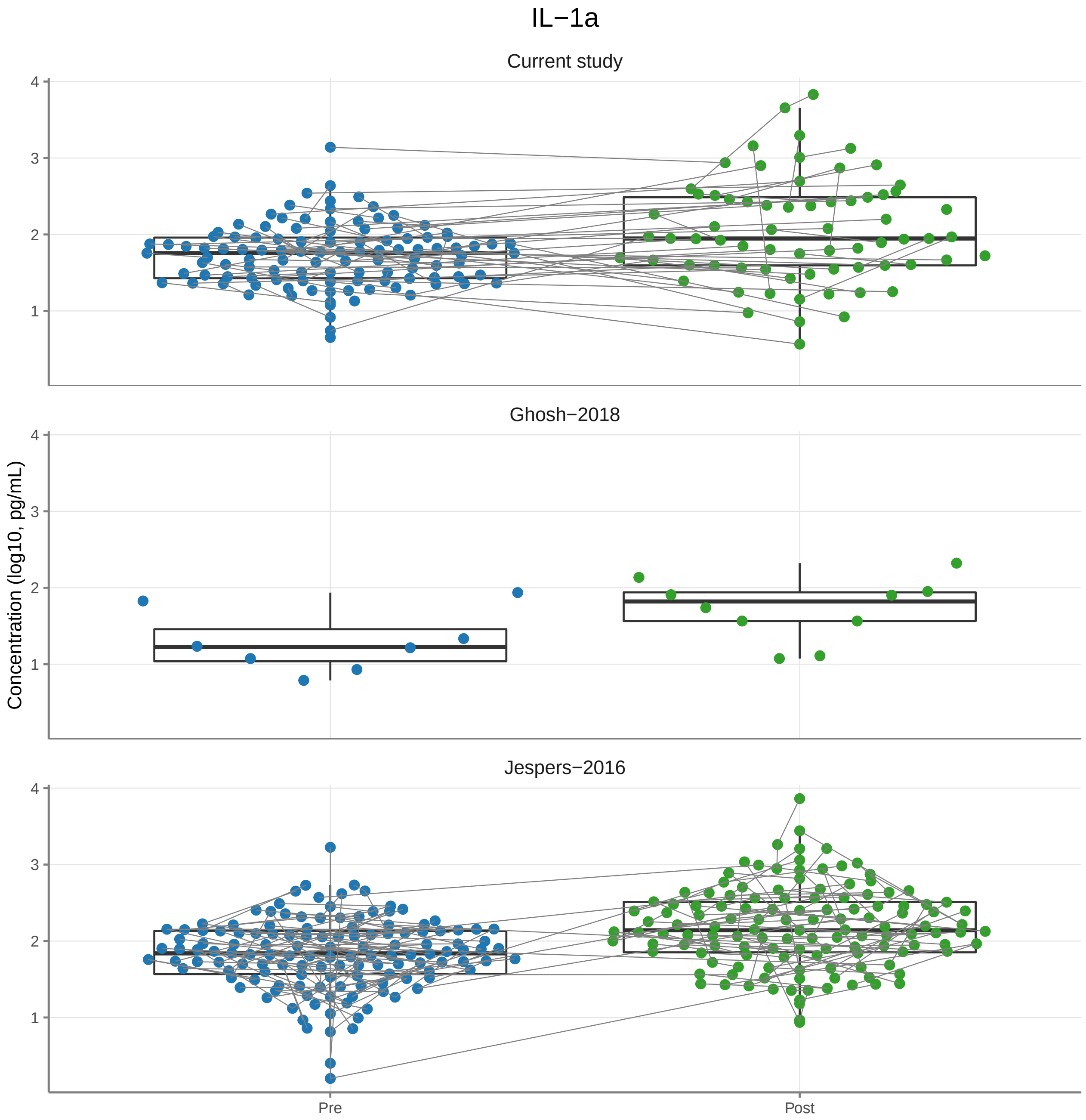
IL−1a
Current study
Ghosh−2018
Jespers−2016
Concentration (log10, pg/mL)
1
2
3
4
Pre
Post

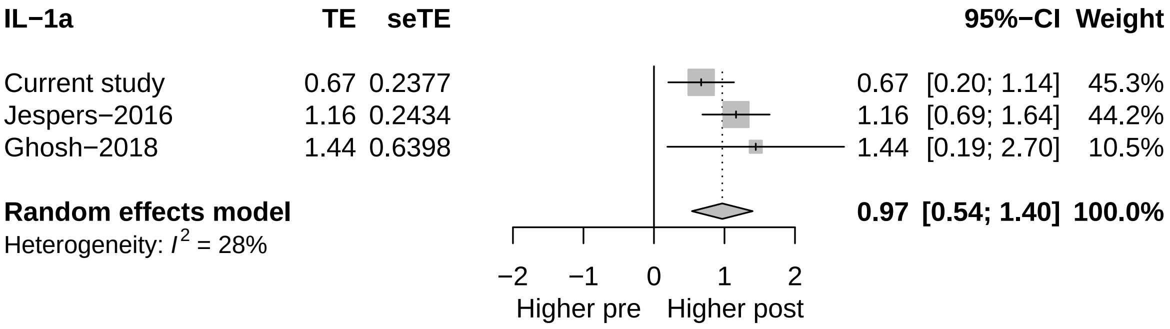
IL−1a
TE
seTE
95%−CI
Weight
Current study 0.67 0.2377 0.67 [0.20; 1.14] 45.3%
Jespers−2016 1.16 0.2434 1.16 [0.69; 1.64] 44.2%
Ghosh−2018 1.44 0.6398 1.44 [0.19; 2.70] 10.5%
Random effects model 0.97 [0.54; 1.40] 100.0%
Heterogeneity: I2 = 28%
−2 −1 0 1 2
Higher pre
Higher post

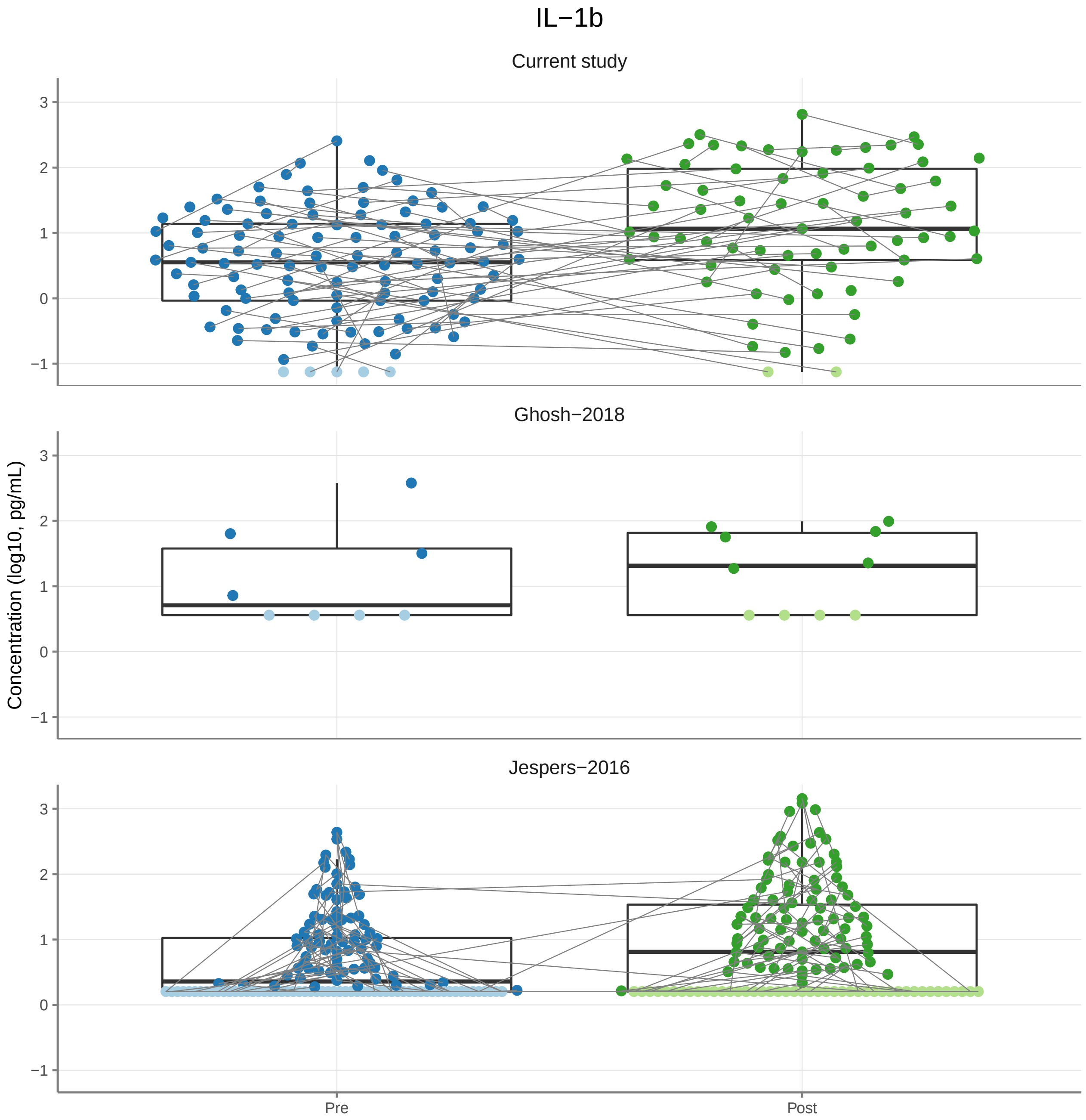
IL−1b
Current study
Ghosh−2018
Jespers−2016
Concentration (log10, pg/mL)
3
2
1
0
−1
Pre
Post

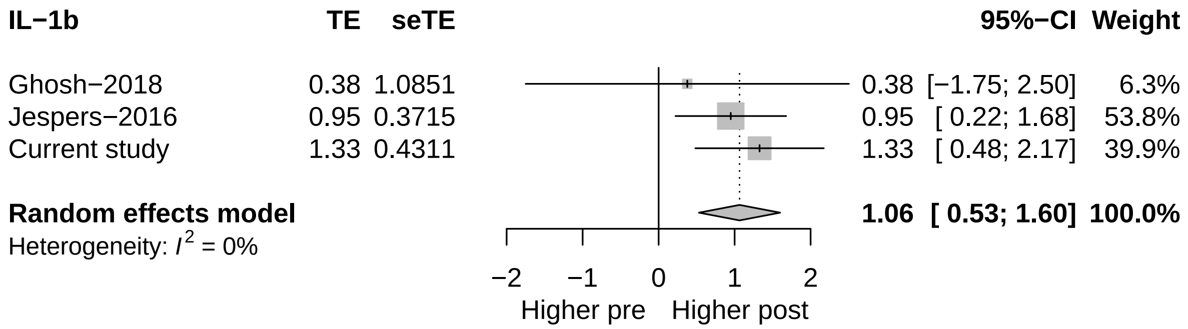
IL−1b
TE
seTE
95%−CI
Weight
Ghosh−2018 0.38 1.0851 0.38 [−1.75; 2.50] 6.3%
Jespers−2016 0.95 0.3715 0.95 [0.22; 1.68] 53.8%
Current study 1.33 0.4311 1.33 [0.48; 2.17] 39.9%
Random effects model 1.06 [0.53; 1.60] 100.0%
Heterogeneity: I^2 = 0%
−2
−1
0
1
2
Higher pre
Higher post

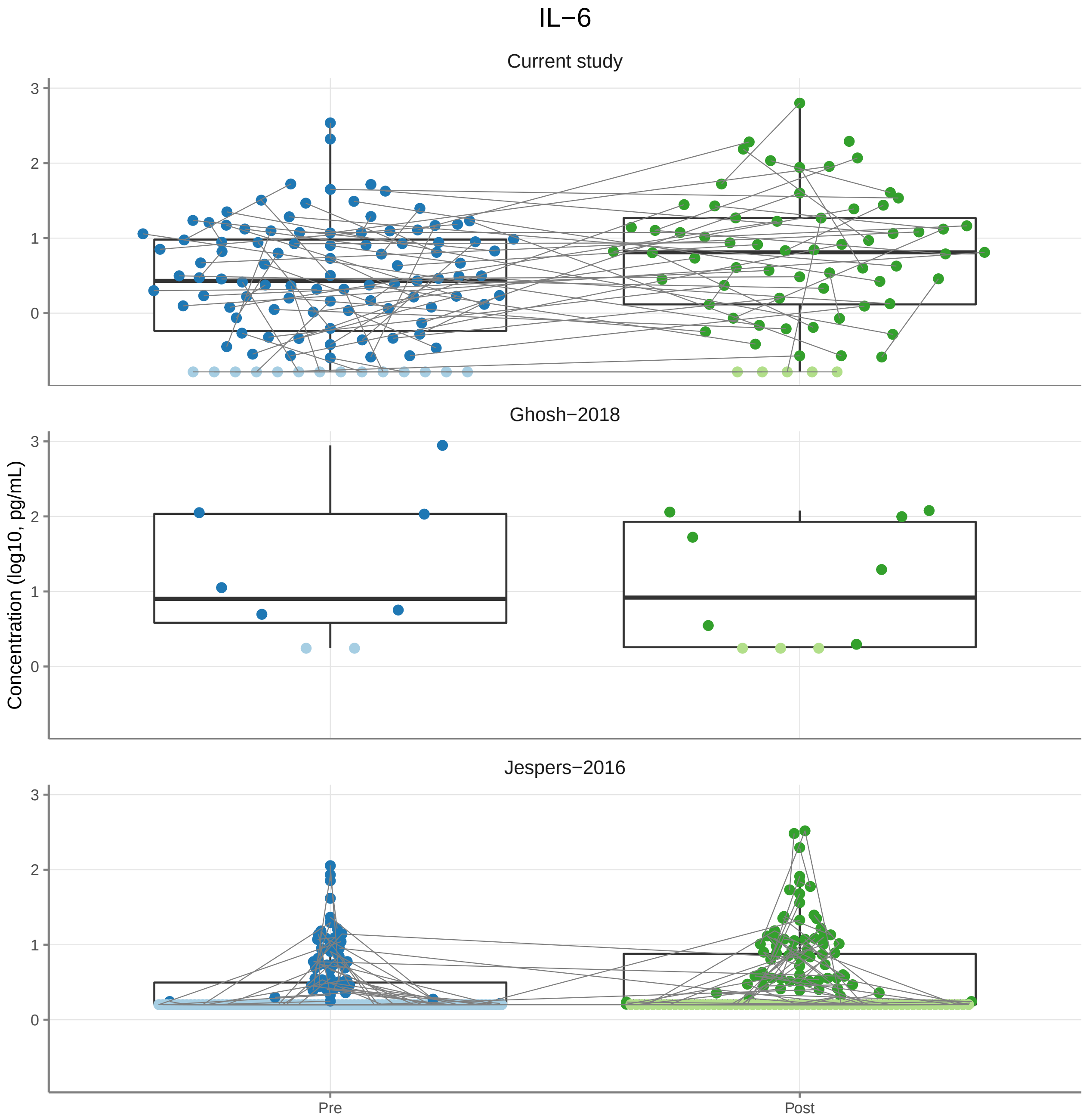
IL−6
Current study
Ghosh−2018
Jespers−2016
Concentration (log10, pg/mL)
3
2
1
0
Pre
Post

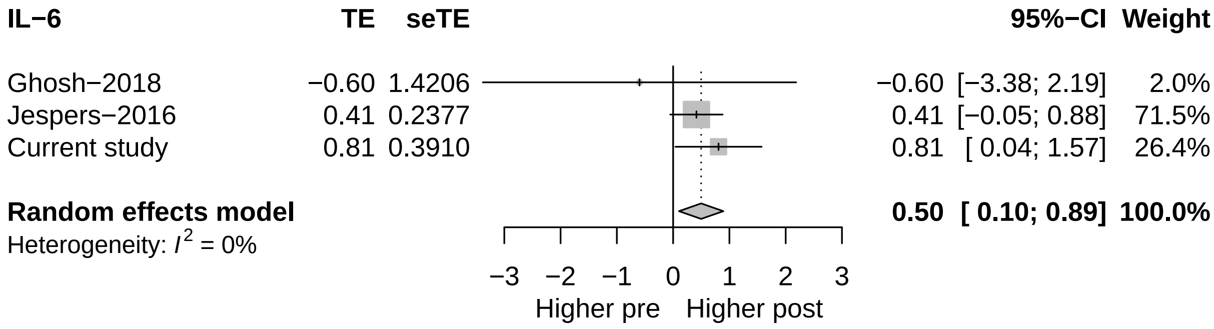
IL−6
TE
seTE
95%−CI
Weight
Ghosh−2018 −0.60 1.4206 −0.60 [−3.38; 2.19] 2.0%
Jespers−2016 0.41 0.2377 0.41 [−0.05; 0.88] 71.5%
Current study 0.81 0.3910 0.81 [0.04; 1.57] 26.4%
Random effects model 0.50 [0.10; 0.89] 100.0%
Heterogeneity: I² = 0%
−3 −2 −1 0 1 2 3
Higher pre
Higher post

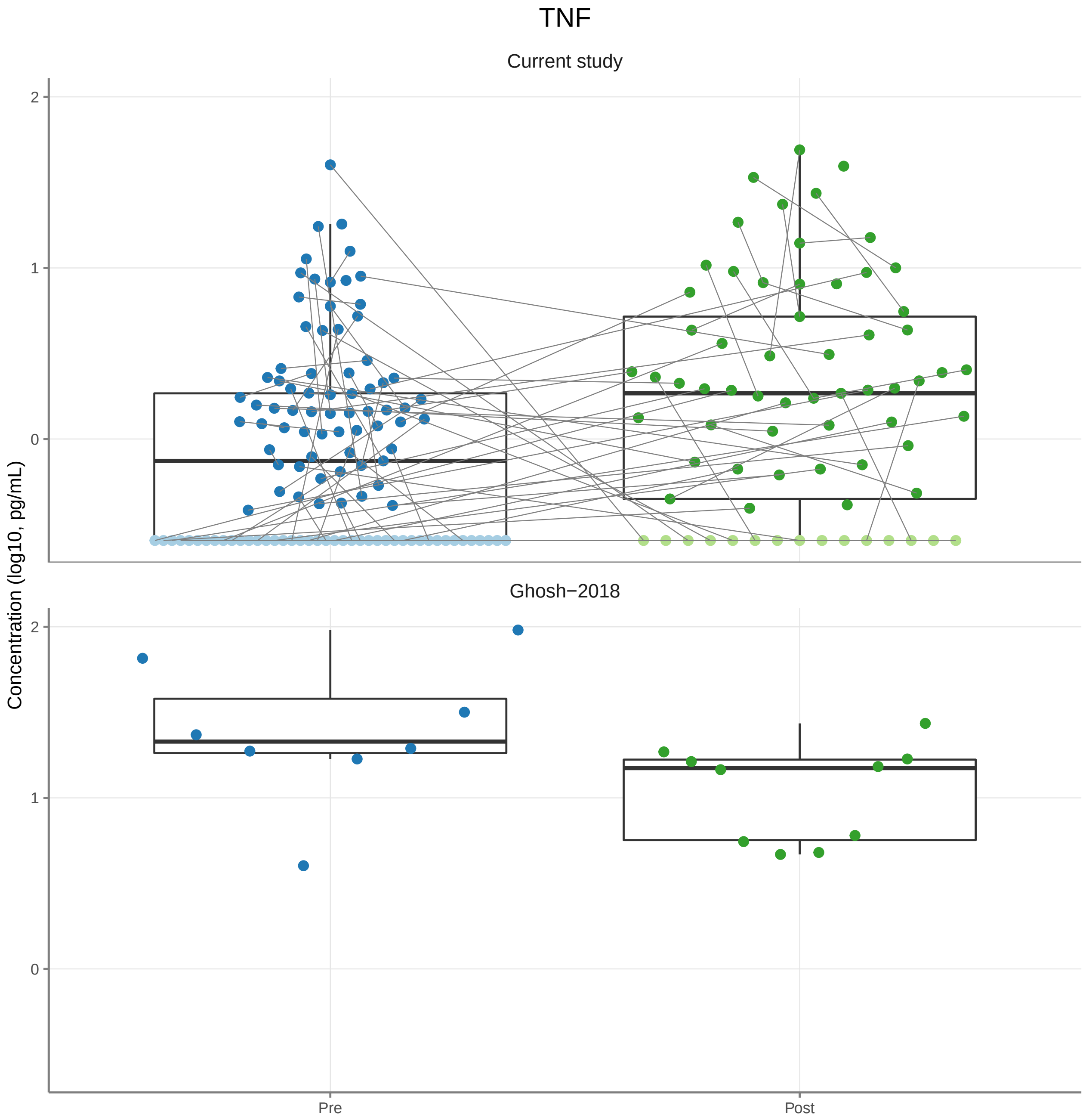
TNF
Current study
Ghosh−2018
Concentration (log10, pg/mL)
2
1
0
2
1
0
Pre
Post

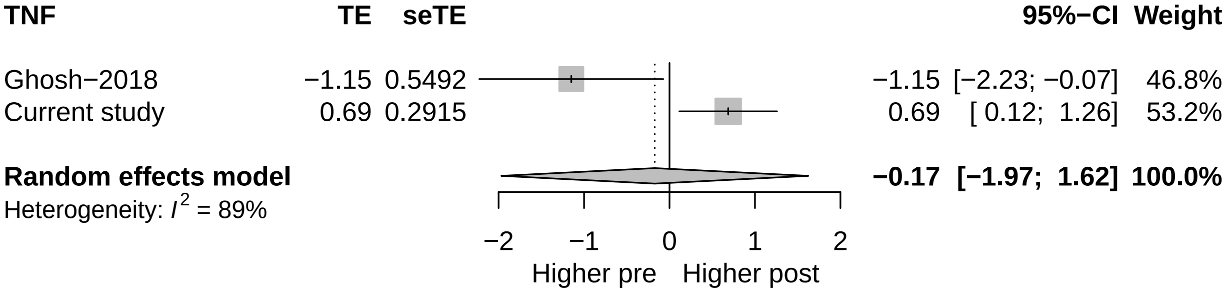
TNF
TE
seTE
95%−CI
Weight
Ghosh−2018 −1.15 0.5492 −1.15 [−2.23; −0.07] 46.8%
Current study 0.69 0.2915 0.69 [0.12; 1.26] 53.2%
Random effects model −0.17 [−1.97; 1.62] 100.0%
Heterogeneity: I² = 89%
−2 −1 0 1 2
Higher pre
Higher post
