# Supplementary material for: Starting to have sexual intercourse is associated with increases in cervicovaginal immune mediators in young women: a prospective study and meta-analysis
Source: eLife. 2022 Oct 25;11:e78565. doi: 10.7554/eLife.78565 (PMC9596159; doi:10.7554/eLife.78565)
Supplement: Source code 1. — An R project which cleans the raw data, runs all of the models reported in the paper, and generates the figures and tables. [file elife-78565-code1.zip › ThikaPrePostSexManuscript/README.html]

README


# README

## Data availability

The raw data files are available in `data/raw/`. These files have a variety of formats between studies.

The cleaned data files are available in `data/clean/`. These files are consistently formatted across studies.

## Reproducing the analysis

The rest of this document describes how to reproduce the data analysis and figures.

All data go into subfolders of this folder.

## Running the analysis

Open `R/source_all.R` and install all of the packages listed there.

Open `make_paper.R`. Assuming the working directory is the same as the directory containing this file, the file paths will all be correct. To reproduce the analysis, source `make_paper.R`. This will run code to:

- Load and clean all of the data files
- Perform statistical analysis
- Generate all figures
- Generate all statistics reported in the manuscript
- Generate the supplementary files

It will take a while to run.

Questions? smhughes@uw.edu

## File and directory summary

- `data/` contains all of the data. The `raw/` sub-directory contains the raw files and the `clean/` subdirectory contains the files after processing. `required_files/` contains other necessary files that are not data.
- `figures/images` contains the figures
- `make_paper.R` runs all the code necessary to reproduce the analysis and generate the functions
- `R/` contains code files
  - `clean_*.R` load and clean raw data
  - `model_data.R` runs the mixed models
  - `meta_analysis.R` cleans the data from the other studies, runs the models on them, and performs the meta-analysis
  - `figures.R` generates the figures
  - `generate_supplement.R` creates the supplementary tables
  - `get_data.R` and `functions_*.R` load helper functions
  - `source_all.R` loads all the packages and helper functions
  - `tables.Rmd` creates statistical tables used in writing the paper
  - `plot_each_analyte_meta-analysis_separately.Rmd` creates several plots for each meta-analysis

## Session info

The analysis for the paper was run using the following versions of R and the packages:

```
## - Session info -----------------------------------------------------------------------------------
##  setting  value                       
##  version  R version 4.0.0 (2020-04-24)
##  os       Windows 10 x64              
##  system   x86_64, mingw32             
##  ui       RTerm                       
##  language (EN)                        
##  collate  English_United States.1252  
##  ctype    English_United States.1252  
##  tz       America/Los_Angeles         
##  date     2022-08-05                  
## 
## - Packages ---------------------------------------------------------------------------------------
##  package      * version    date       lib source        
##  assertthat     0.2.1      2019-03-21 [1] CRAN (R 4.0.0)
##  backports      1.1.6      2020-04-05 [1] CRAN (R 4.0.0)
##  beeswarm       0.2.3      2016-04-25 [1] CRAN (R 4.0.0)
##  boot           1.3-24     2019-12-20 [2] CRAN (R 4.0.0)
##  broom          0.7.8      2021-06-24 [1] CRAN (R 4.0.5)
##  cellranger     1.1.0      2016-07-27 [1] CRAN (R 4.0.0)
##  cli            3.0.0      2021-06-30 [1] CRAN (R 4.0.5)
##  colorspace     1.4-1      2019-03-18 [1] CRAN (R 4.0.0)
##  CompQuadForm   1.4.3      2017-04-12 [1] CRAN (R 4.0.0)
##  crayon         1.3.4      2017-09-16 [1] CRAN (R 4.0.0)
##  DBI            1.1.0      2019-12-15 [1] CRAN (R 4.0.0)
##  dbplyr         1.4.3      2020-04-19 [1] CRAN (R 4.0.0)
##  digest         0.6.25     2020-02-23 [1] CRAN (R 4.0.0)
##  dplyr        * 1.0.7      2021-06-18 [1] CRAN (R 4.0.5)
##  ellipsis       0.3.2      2021-04-29 [1] CRAN (R 4.0.5)
##  evaluate       0.14       2019-05-28 [1] CRAN (R 4.0.0)
##  fansi          0.4.1      2020-01-08 [1] CRAN (R 4.0.0)
##  forcats      * 0.5.0      2020-03-01 [1] CRAN (R 4.0.0)
##  fs             1.5.0      2020-07-31 [1] CRAN (R 4.0.5)
##  generics       0.1.0      2020-10-31 [1] CRAN (R 4.0.5)
##  ggbeeswarm   * 0.6.0      2017-08-07 [1] CRAN (R 4.0.0)
##  ggplot2      * 3.3.5      2021-06-25 [1] CRAN (R 4.0.5)
##  glue           1.4.0      2020-04-03 [1] CRAN (R 4.0.0)
##  gtable         0.3.0      2019-03-25 [1] CRAN (R 4.0.0)
##  gtsummary    * 1.3.0      2020-04-17 [1] CRAN (R 4.0.0)
##  haven          2.2.0      2019-11-08 [1] CRAN (R 4.0.0)
##  here         * 0.1        2017-05-28 [1] CRAN (R 4.0.0)
##  hms            0.5.3      2020-01-08 [1] CRAN (R 4.0.0)
##  htmltools      0.5.1.1    2021-01-22 [1] CRAN (R 4.0.5)
##  httr           1.4.2      2020-07-20 [1] CRAN (R 4.0.5)
##  janitor      * 2.0.1      2020-04-12 [1] CRAN (R 4.0.0)
##  jsonlite       1.6.1      2020-02-02 [1] CRAN (R 4.0.0)
##  knitr          1.28       2020-02-06 [1] CRAN (R 4.0.0)
##  lattice        0.20-41    2020-04-02 [2] CRAN (R 4.0.0)
##  lifecycle      1.0.0      2021-02-15 [1] CRAN (R 4.0.5)
##  lme4         * 1.1-23     2020-04-07 [1] CRAN (R 4.0.0)
##  lmerTest     * 3.1-2      2020-04-08 [1] CRAN (R 4.0.0)
##  lubridate      1.7.8      2020-04-06 [1] CRAN (R 4.0.0)
##  magrittr       1.5        2014-11-22 [1] CRAN (R 4.0.0)
##  MASS           7.3-51.5   2019-12-20 [2] CRAN (R 4.0.0)
##  mathjaxr       1.4-0      2021-03-01 [1] CRAN (R 4.0.5)
##  Matrix       * 1.3-4      2021-06-01 [1] CRAN (R 4.0.5)
##  meta         * 5.0-1      2021-10-20 [1] CRAN (R 4.0.5)
##  metafor        3.0-2      2021-06-09 [1] CRAN (R 4.0.5)
##  minqa          1.2.4      2014-10-09 [1] CRAN (R 4.0.0)
##  modelr         0.1.7      2020-04-30 [1] CRAN (R 4.0.0)
##  munsell        0.5.0      2018-06-12 [1] CRAN (R 4.0.0)
##  nlme           3.1-147    2020-04-13 [2] CRAN (R 4.0.0)
##  nloptr         1.2.2.1    2020-03-11 [1] CRAN (R 4.0.0)
##  numDeriv       2016.8-1.1 2019-06-06 [1] CRAN (R 4.0.0)
##  pander       * 0.6.3      2018-11-06 [1] CRAN (R 4.0.0)
##  patchwork    * 1.0.0      2019-12-01 [1] CRAN (R 4.0.0)
##  pillar         1.6.1      2021-05-16 [1] CRAN (R 4.0.5)
##  pkgconfig      2.0.3      2019-09-22 [1] CRAN (R 4.0.0)
##  purrr        * 0.3.4      2020-04-17 [1] CRAN (R 4.0.0)
##  R6             2.4.1      2019-11-12 [1] CRAN (R 4.0.0)
##  Rcpp           1.0.7      2021-07-07 [1] CRAN (R 4.0.5)
##  readr        * 1.3.1      2018-12-21 [1] CRAN (R 4.0.0)
##  readxl       * 1.3.1      2019-03-13 [1] CRAN (R 4.0.0)
##  reprex         0.3.0      2019-05-16 [1] CRAN (R 4.0.0)
##  rlang          0.4.11     2021-04-30 [1] CRAN (R 4.0.5)
##  rmarkdown    * 2.1        2020-01-20 [1] CRAN (R 4.0.0)
##  rprojroot      1.3-2      2018-01-03 [1] CRAN (R 4.0.0)
##  rstudioapi     0.13       2020-11-12 [1] CRAN (R 4.0.5)
##  rvest          0.3.5      2019-11-08 [1] CRAN (R 4.0.0)
##  scales         1.1.1      2020-05-11 [1] CRAN (R 4.0.0)
##  sessioninfo    1.1.1      2018-11-05 [1] CRAN (R 4.0.0)
##  snakecase      0.11.0     2019-05-25 [1] CRAN (R 4.0.0)
##  statmod        1.4.34     2020-02-17 [1] CRAN (R 4.0.0)
##  stringi        1.4.6      2020-02-17 [1] CRAN (R 4.0.0)
##  stringr      * 1.4.0      2019-02-10 [1] CRAN (R 4.0.0)
##  tibble       * 3.1.2      2021-05-16 [1] CRAN (R 4.0.5)
##  tidyr        * 1.1.3      2021-03-03 [1] CRAN (R 4.0.5)
##  tidyselect     1.1.0      2020-05-11 [1] CRAN (R 4.0.0)
##  tidyverse    * 1.3.0      2019-11-21 [1] CRAN (R 4.0.0)
##  utf8           1.1.4      2018-05-24 [1] CRAN (R 4.0.0)
##  vctrs          0.3.8      2021-04-29 [1] CRAN (R 4.0.5)
##  vipor          0.4.5      2017-03-22 [1] CRAN (R 4.0.0)
##  withr          2.4.2      2021-04-18 [1] CRAN (R 4.0.5)
##  xfun           0.13       2020-04-13 [1] CRAN (R 4.0.0)
##  xml2           1.3.2      2020-04-23 [1] CRAN (R 4.0.0)
##  yaml           2.2.1      2020-02-01 [1] CRAN (R 4.0.0)
## 
## [1] C:/Users/smhughes/Documents/R/win-library/4.0
## [2] C:/Program Files/R/R-4.0.0/library
```
